# The Philosophy of Marriage, in the Social, Moral, and Physical Relations; with an Account of the Diseases of the Genito-Urinary Organs, Which Impair or Destroy the Reproductive Function, and Induce a Variety of Complaints; with the Physiology of Generation in the Vegetable and Animal Kingdoms; Being Part of a Course of Obstetric Lectures Delivered at the North London School of Medicine, Etc.

**Published:** 1848-11

**Authors:** 


*The Philosophy of Marriage, in the social, moral, and physical relations; with an account of the diseases of the Genito-Urinary Organs, which impair or destroy the reproductive function, and induce a variety of complaints; with the physiology of generation in the Vegetable and Animal Kingdoms; being part of a course of Obstetric Lectures delivered at the North London School of Medicine, etc.* By MICHAEL RYAN, M. D., etc. etc. *From the last London Edition.* 18mo. pp. 285. Barrington & Haswell. Philadelphia, 1848.

Dr. Ryan is the author of several works on medical subjects, in all of which we find his peculiar traits as a writer—learned, acute, often eccentric in his opinions and modes of expression, and never remarkable for order.

The present work has passed through four editions in London, which is some proof of its popularity: but whether its patrons have been members of the profession, or its circulation has been less restricted, as would seem from its character to have been the design of the author, we are not advised. For laymen it contains wise counsel as well as valuable information, but it also contains a great deal that is unfit for the eye of an uneducated reader. Members of the medical profession will find in it much that is curious and entertaining, as rare cases, anecdotes, recondite opinions of eminent men, interspersed with useful medical instruction.

About two years ago, a garbled edition of this work was published in the city of New York, under the title of "Mysteries of Marriage," with an obscene frontispiece, and a *Preface* (American) in full character with the licentious engraving. Of course only such parts were embodied in that edition as were likely to minister to the depraved curiosity of the class of readers for which the edition was designed. The present edition, as any one may suppose from the character of the publishers, is free from these objections. It is a reprint of the work as put forth by the author, without abridgement, note, or addition of any kind. Still, from its many objectionable features, we cannot help regretting that it should have been honoured by a republication in any shape.

